# Structure of the Human Factor VIII C2 Domain in Complex with the 3E6 Inhibitory Antibody

**DOI:** 10.1038/srep17216

**Published:** 2015-11-24

**Authors:** Michelle E. Wuerth, Rebecca K. Cragerud, P. Clint Spiegel

**Affiliations:** 1 Western Washington University, Department of Chemistry, 516 High Street, Bellingham, WA 98225-9150

## Abstract

Blood coagulation factor VIII is a glycoprotein cofactor that is essential for the intrinsic pathway of the blood coagulation cascade. Inhibitory antibodies arise either spontaneously or in response to therapeutic infusion of functional factor VIII into hemophilia A patients, many of which are specific to the factor VIII C2 domain. The immune response is largely parsed into “classical” and “non-classical” inhibitory antibodies, which bind to opposing faces cooperatively. In this study, the 2.61 Å resolution structure of the C2 domain in complex with the antigen-binding fragment of the 3E6 classical inhibitory antibody is reported. The binding interface is largely conserved when aligned with the previously determined structure of the C2 domain in complex with two antibodies simultaneously. Further inspection of the B factors for the C2 domain in various X-ray crystal structures indicates that 3E6 antibody binding decreases the thermal motion behavior of surface loops in the C2 domain on the opposing face, thereby suggesting that cooperative antibody binding is a dynamic effect. Understanding the structural nature of the immune response to factor VIII following hemophilia A treatment will help lead to the development of better therapeutic reagents.

Hemophilia A is a blood clotting disorder caused by a lack of functional blood coagulation factor VIII (fVIII), a protein cofactor essential to the intrinsic pathway of the blood clotting cascade. Congenital hemophilia A, which varies in severity depending on the amount of functional fVIII present, is an X-linked disorder affecting 1 in 5,000 males worldwide^1^. The primary treatment for the disease is therapeutic infusions of recombinant fVIII, either in an acute or prophylactic manner^2^,^3^. The most significant complication to this treatment is the development of neutralizing inhibitory antibodies directed against the infused fVIII. Approximately 30% of patients receiving replacement therapy develop inhibitory antibodies, an immune response leading to the clearance of fVIII from circulation and continued lack of clotting function^4^,^5^,^6^.

Coagulation fVIII is a 2,332-residue glycoprotein that is expressed with the domain arrangement of A1-A2-B-*a3*-A3-C1-C2 prior to proteolytic processing^7^,^8^,^9^,^10^. The fVIII protein circulates in the bloodstream in its inactive form as a heterodimer, consisting of a heavy chain (A1-A2-B) and a light chain (*a3*-A3-C1-C2)^11^. The heterodimeric form of fVIII is noncovalently bound to the glycoprotein von Willebrand factor (vWF), an interaction preventing the breakdown of fVIII in circulation^12^,^13^,^14^. Following vascular damage, fVIII undergoes proteolytic activation by thrombin or factor Xa (fXa) to form a heterotrimer (A1/A2/A3-C1-C2) that dissociates from vWF and binds to activated platelet surfaces, where it serves as a cofactor for the serine protease factor IXa^15^,^16^. This complex, known as the intrinsic “tenase” complex, is responsible for converting fX to fXa at an increased rate of approximately 200,000-fold^16^,^17^.

Characterization of the immune response to fVIII has revealed that the A2 and C2 domains harbor the majority of epitopes recognized by inhibitory antibodies against fVIII^18^,^19^,^20^,^21^. Antibodies that garner specificity for the fVIII C2 domain have previously been categorized into two classes based on differing mechanisms of fVIII inhibition^20^. “Classical” antibody inhibitors are defined as inhibitors that block the binding of fVIII to vWF or phospholipid surfaces, which have been demonstrated to involve partially overlapping sites on the C2 domain^14^,^22^,^23^,^24^. By contrast, “non-classical” antibody inhibitors block the proteolytic activation of fVIII by thrombin or fXa, thus preventing the dissociation of fVIII from vWF^20^,^25^,^26^. For comparison, the non-classical antibodies comprise the majority of fVIII C2 domain inhibitors with high titers relative to the classical antibodies, which are commonly more pathogenic^20^,^26^.

Detailed structural analyses of the fVIII C2 domain in complex with inhibitory antibodies have allowed for the characterization of both classical and non-classical epitopes^27^,^28^,^29^. Initial structural studies of a high titer classical antibody inhibitor, BO2C11, in complex with the fVIII C2 domain indicated that the BO2C11 epitope significantly overlapped with the region proposed to be involved in membrane binding, which was completely sequestered upon complex formation^27^. Additionally, a more recent X-ray crystal structure of the fVIII C2 domain bound simultaneously in a ternary complex to antigen binding fragments (F_AB_s) of both a classical (3E6) and non-classical (G99) antibody demonstrated that these inhibitors bind to opposite sides of the C2 domain with minimal overlap with the BO2C11 epitope^28^. Moreover, the classical antibody 3E6 was shown to occlude residues implicated in vWF binding^30^, while the epitope of non-classical antibody G99 included residues involved in binding both factors IXa and Xa^31^,^32^,^33^. Previous binding data have suggested that several classical/non-classical antibody pairs bind cooperatively to fVIII^34^,^35^, but no structural evidence for the observed cooperativity has been determined. In this study, we present the X-ray crystal structure to 2.6 Å resolution of the fVIII C2 domain in a binary complex with the F_AB_ of the classical antibody 3E6. Comparisons between the C2 domain/3E6 F_AB_ binary structure with the C2 domain/3E6 F_AB_/G99 F_AB_ ternary structure illustrate the structural conservation and dynamic behavior of C2 domain epitopes for classical antibody inhibitors in the presence and absence of non-classical antibody inhibitors.

## Results and Discussion

### Overall structure

**Figure 1:**
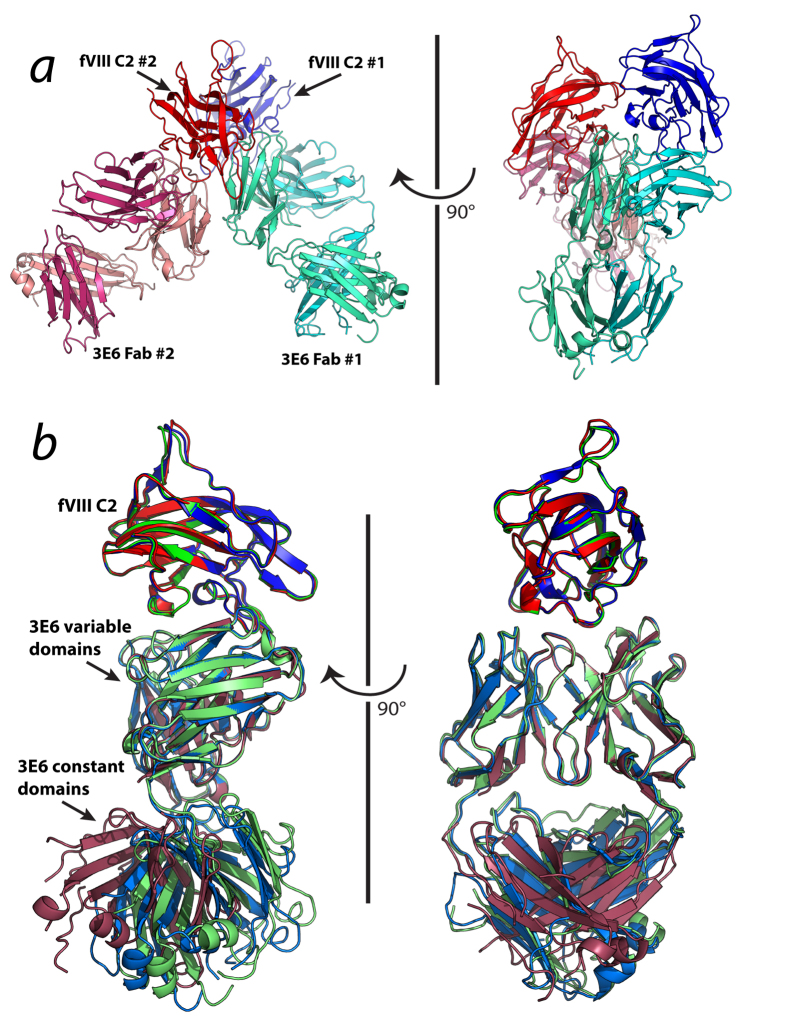
Ribbon diagram presentation of the factor VIII C2 domain/3E6 F_AB_ complex. (***a***) The asymmetric unit of the C2 domain/3E6 F_AB_ complex contains two copies of the biological unit. Blue/red: fVIII C2 domain from complexes 1 and 2, respectively; cyan/green cyan: light and heavy chains of 3E6 F_AB_ from complex 1, respectively; magenta/salmon: light and heavy chains of 3E6 F_AB_ from complex 2, respectively. (***b***) Superposition of C2 domain/3E6 complexes 1 and 2 with the C2/3E6 component of the C2/3E6/G99 ternary complex structure^28^. The alignment was limited to the C2 domain and variable domain structures. Green: C2/3E6 from the ternary complex; blue: C2/3E6 from binary complex 1, red: C2/3E6 from binary complex 2.

The structure of the fVIII C2 domain in complex with the inhibitory antibody F_AB_, 3E6, was refined to a resolution of 2.61 Å with a final R_work_ and R_free_ of 19.9% and 26.7%, respectively (Table 1). Iterative molecular replacement was performed with Phaser, which consisted of independent searches for two copies each of the fVIII C2 domain, the variable domain dimer and the constant domain dimer. The asymmetric unit contains two C2 domain/3E6 F_AB_ complexes, which were built separately due to structural changes in the F_AB_ elbow angle (Fig. 1a). Validation of the refined structure indicated 92% of residues are within the favored Ramachandran region (Table 1). The structure of each fVIII C2 domain is well resolved, consisting of a continuous chain trace from residues Cys2174- Glu2327 (chains M and G for complexes 1 and 2, respectively). The 3E6 F_AB_ from complex 1 (chains A and B for the antibody heavy and light chains, respectively) was built to represent a complete model with the exception of residues 132–133 from chain A, which reside in a loop at the base of the constant domain. By contrast, the F_AB_ from complex 2 possessed poorly resolved density for the constant domains of both the heavy and light chains (chains E and F, respectively). Within the F_AB_ constant domains of complex 2, residues 177 and 130–135 were absent from the heavy chain and residues 106, 149–154, 187–191 and 198 203 were absent from the light chain. Regardless of this region of poorly defined density, the fVIII C2 domain/3E6 F_AB_ interface was well defined by unambiguous electron density for both the C2 domain as well as the CDR loops for each complex.

The overall structure of the fVIII C2 domain/3E6 F_AB_ binding interface is highly conserved. Upon superposition of the C2 domain and variable domains for each binary complex with the C2/3E6 portion of the previously determined structure of the C2 domain/3E6/G99 F_AB_ ternary complex^28^, the RMSD for complexes 1 and 2 were calculated to be 0.328 and 0.383 for Cα atoms, respectively (Fig. 1b). The most significant deviation in the C2/3E6 binary crystal structure was present at the elbow angle between the variable and constant domains of each complex. Specifically, the C2/3E6 portion of the ternary complex displayed the most extended structure, with an elbow angle close to zero. By contrast, complexes 1 and 2 possessed increasing deviations from planarity about the F_AB_ elbow, respectively (Fig. 1b). While this discrepancy is notable, changes in elbow angles for F_AB_ structures are often present and likely do not contribute significantly to the observed cooperativity for anti-C2 domain antibody binding^36^.

**Figure 2:**
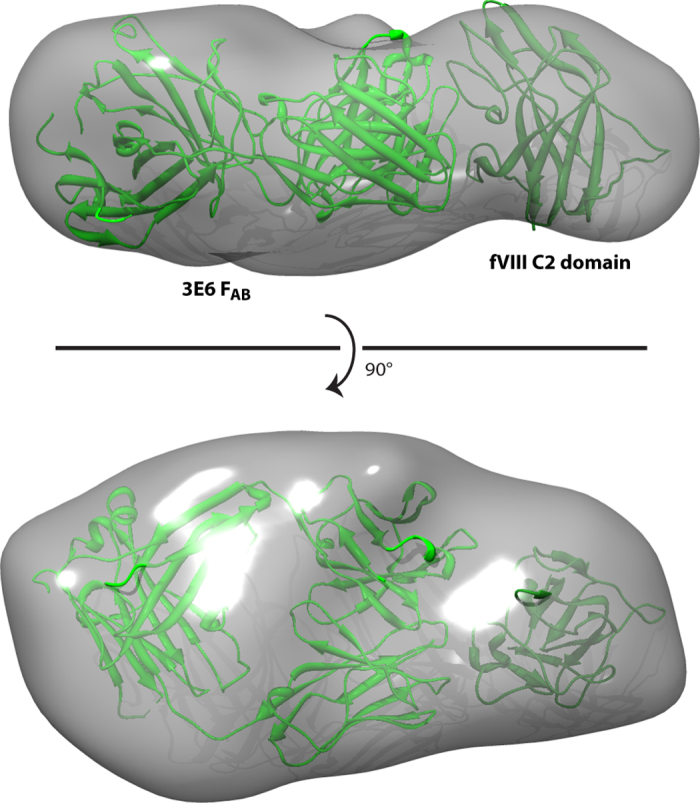
SAXS envelope of the factor VIII C2 domain/3E6 F_AB_ complex. Based on previous SAXS data^29^, molecular envelopes were calculated with DAMMIF, averaged with DAMAVER and refined with DAMMIN. Rigid body modeling of the C2 domain/3E6 complex from the C2/3E6/G99 ternary structure^28^ was modeled as a rigid body into the SAXS envelope with the “Fit in Map” algorithm in Chimera.

To further understand the solution conformation of the 3E6 F_AB_ in complex with the fVIII C2 domain, each C2/3E6 binary structure was fit into a newly calculated SAXS envelope of the C2/3E6 complex from previously collected SAXS data^29^. Subsequent to manual alignment of each structure with the SAXS envelope, the ‘Fit in Map’ algorithm in Chimera was employed to optimize the alignment and calculate a correlation coefficient. While all the models fit within reason to the SAXS envelope, the C2/3E6 structures from binary complex 1 and the ternary complex yielded the highest correlation (>0.97), indicating that the solution conformation of the C2/3E6 complex is more extended with an F_AB_ elbow angle approaching 180° (Fig. 2).

### The factor VIII C2 domain/3E6 F_AB_ binding interface

**Figure 3:**
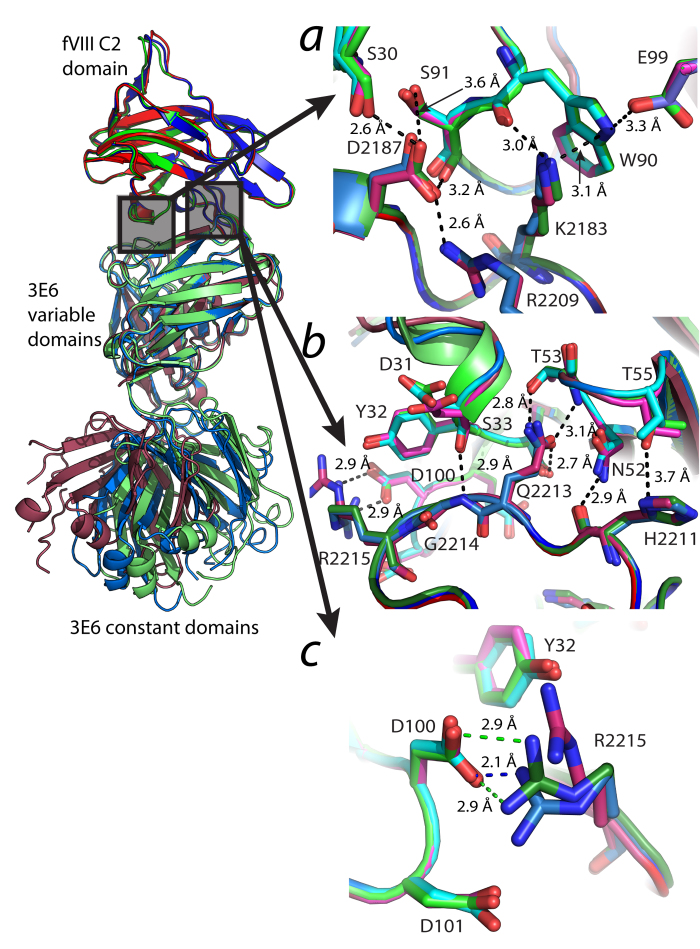
The factor VIII C2 domain/3E6 F_AB_ binding interface. (***a***) The Glu2181-Ala2188 loop. Both Lys2183 and Asp2187 form conserved interactions with the 3E6 variable domain. (***b***) The Thr2202-Arg2215 loop. Conserved interactions are present for His2211, Gln2213 and Gly2214. (***c***) Conformational heterogeneity for Arg2215 in different C2/3E6 complexes. Carbon color labeling: green: C2/3E6 from the ternary complex; blue/cyan: C2/3E6 from binary complex 1, red/magenta: C2/3E6 from binary complex 2.

The 3E6 antibody binding epitope in the fVIII C2 domain is highly conserved amongst the two binary complexes determined in this study along with the previously characterized epitope from the C2 domain/3E6/G99 F_AB_ ternary complex^28^. For each binary complex, all residues proximal to the binding interface are fit within defined electron density (Supplementary Figure S1). The extent of buried surface area between the two binary complexes and the C2/3E6 components of the ternary complex are not significantly different. The C2 domain epitope consists of two loops, Glu2181-Ala2188 and Thr2202-Arg2215 (Fig. 3a,b). Backbone conformations of resdiues proximal to the binding interface do not change significantly, as is the same for the sidechains of His2211, Gln2213, Lys2183, Arg2209 and Asp2187, all of which contribute directly to the 3E6-binding interface. Conformational changes are present, however, for the sidechain of Arg2215 (Fig. 3c). In the ternary structure, Arg2215 makes an optimal salt bridge with Asp100 of the 3E6 heavy chain. In contrast to this interaction, the C2/3E6 binary complex 1 indicates a single hydrogen bond between Arg2215 and Asp100 while complex 2 shows Arg2215 to be projecting away from D100, out of hydrogen bonding distance.

Following the characterization of each binding interface for both binary complexes and comparing them to the ternary complex, it was concluded that the mechanism for cooperativity between classical and non-classical anti-fVIII C2 domain inhibitory antibodies is likely not due to significant changes in conformation directly at the binding interface. Given that cooperativity is observed for several classical/non-classical antibody pairs, we hypothesized that the cooperative behavior may be due to either electrostatic or dynamic perturbation. Following pKa calculations with PROPKA for the fVIII C2 domain in isolation as well as in complex with the 3E6 antibody, it was observed that the majority of significant pKa perturbations occurred directly at the 3E6 interface, as expected. Specifically, residues with significant pKa perturbations (>0.25 pH units) in the region of the 3E6 epitope were Glu2181, Glu2322, Lys2183, Lys2207, Lys2236, His2211, Arg2209, Arg2215 and Arg2320. By contrast, two residues at or near the non-classical G99 F_AB_ epitope with significant pKa perturbations were His2269 (−0.4 pH units) and His2315 (−0.6 pH units).

**Figure 4:**
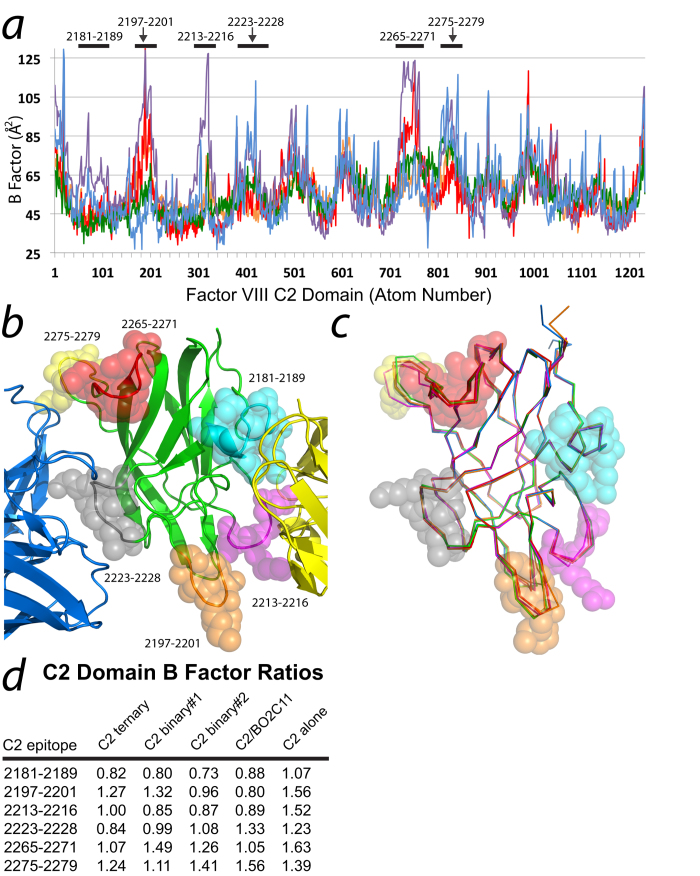
Analysis of B factors for different factor VIII C2 domain complexes. (***a***) Normalized plot of B factors for all atoms in each C2 domain structure. Red: C2/3E6 F_AB_ binary complex 1 (pdb#: 4XZU); green: C2/3E6 F_AB_ binary complex 2 (pdb#: 4XZU); orange: the C2/3E6/G99 F_AB_ ternary complex (pdb#: 4KI5); blue: the C2/BO2C11 F_AB_ complex (pdb#: 1IQD); purple: the isolated fVIII C2 domain (pdb#: 1D7P). (***b***) Structural representation of loops with different B factors relative to overall average B factors for each complex. VdW spheres: defined loops as defined in (***d***); green: factor VIII C2 domain; yellow: 3E6 variable domain; blue: G99 variable domain. (***c***) C-alpha ribbon representation of the superposition for each fVIII C2 domain in this study (blue: isolated C2 domain, orange: C2/BO2C11 complex, green: C2/G99/3E6 ternary complex, red: C2/3E6 binary #1, magenta: C2/3E6 binary #2). (***d***) B factor ratios for surface loops of the factor VIII C2 domain that possess differential B factors relative to entire C2 domain structure. The ratios are defined as the average B factor for a given loop divided by the overall average B factor for each respective C2 domain structure from a given complex.

To assess the dynamic behavior of the non-classical anti-C2 domain epitopes in the presence and absence of the classical inhibitory antibodies, crystallographic B factors were compared for each C2/3E6 binary complex described herein (pdb#: 4XZU), the C2/3E6/G99 F_AB_ ternary complex (pdb#: 4KI5), the C2/BO2C11 F_AB_ complex (pdb#: 1IQD) and the isolated fVIII C2 domain (pdb#: 1D7P). While overall B factors are largely crystal-dependent^37^, the trend throughout a protein structure should be conserved across different crystal forms as an indication of dynamic behavior in surface loops and protein core rigidity. Thus, normalization of the B factors for all atoms within the fVIII C2 domain structures from each of the aforementioned complexes indicates that general B factor trends are similar for all complexes with a few notable exceptions, which are described in more detail below (Fig. 4). In order to directly compare distinct regions of flexibility across structures of the fVIII C2 domain, localized B factors for each loop were averaged and then divided by the average B factors for the entire C2 domain for each respective structure, which results in a ratio that describes the deviation of localized B factor values relative to each corresponding protein structure. To demonstrate the effectiveness of this comparative analysis, two loops directly at the 3E6 interface indicate specific decreases in B factors due to complex formation (Fig. 4b). Specifically, the Gln2213-Ser2216 loop possesses B-factor decreased ratios for each C2 domain in complex with the 3E6 antibody with an average of 0.91 (1.00, 0.85 and 0.87 for the ternary, binary 1 and binary 2 complexes, respectively). Moreover, the 2213–2216 loop contributes to the epitope in the BO2C11 complex and displays a similar ratio of 0.88. By contrast, the isolated C2 domain possesses a significantly higher B factor ratio of 1.50 (Fig. 4d). The second major loop contributing to the 3E6 epitope is Glu2181-Glun2189, which displays B factor ratios of 0.82, 0.80, 0.73, 0.88 and 1.07 for the ternary, binary 1, binary 2, BO2C11 complexes and the isolated C2 domain, respectively, showing that B factors are generally higher for the isolated C2 domain in contrast to the C2 domain in complex with classical antibodies. Interestingly, three loops that are localized to the G99 epitope also display decreased B factor ratios for the two C2/3E6 binary complexes (Ser2265-Trp2271, Phe2275-Lys2279 and Val2223-Glu2228). Significantly, the 2223–2228 loop contains Lys2227, which represents the strongest binding determinant for the non-classical G99 antibody^20^,^29^. Calculated B factor ratios for the C2/3E6/G99 ternary complex and C2/3E6 binary complexes 1 and 2 were 0.83, 0.99 and 1.08, respectively, while the B factor ratio for this loop in the isolated C2 domain was 1.23 (Fig. 4d). Lastly, it is notable that the Thr2197-Ala2201 loop also possesses lower B factor ratios relative to the isolated C2 domain structure. While this loop does not make direct interactions with either the 3E6 or G99 epitope, it is a β–hairpin loop that presents solvent-exposed hydrophobic residues that bridges both 3E6 and G99 epitopes^29^, is a major component of the BO2C11 epitope^27^, and is hypothesized to be the site of membrane binding^38^,^39^,^40^. Moreover, previous H/D exchange data indicate that the 2197–2201 loop has increased protection factors upon 3E6 binding^41^. As a control, the X-ray crystal structures of each C2 domain in this study were superimposed to illustrate the overall structure of each loop in question has the same or similar conformation across all five structures (Fig. 4c). Taken together, these data suggest that the binding of the 3E6 antibody serves to decrease the dynamic mobility of not only the direct 3E6 epitope, but also various loops either adjacent or on the opposing side of the fVIII C2 domain structure, thus potentially decreasing the entropic cost to antibody binding on the non-classical face^42^,^43^.

## Conclusions

In this study, we have determined the X-ray crystal structure of a classical anti-fVIII C2 domain inhibitory antibody (3E6) in complex with the C2 domain from human blood coagulation factor VIII to 2.61 Å resolution. Inhibitory antibodies often arise following fVIII “replacement therapy” in hemophilia A patients, causing a significant clinical complication of uncontrolled bleeding. Previous antibody binding data indicate that classical and non-classical anti-fVIII antibodies bind cooperatively, but the molecular mechanism of this behavior has not been described. Upon comparison of the two fVIII C2 domain/3E6 F_AB_ complexes within this crystal form with the previously determined C2 domain/3E6/G99 F_AB_ ternary complex illustrate the high level of structural conservation at the binding interface with the exception of Arg2215, which shows different conformers for each complex. Provided that significant structural changes that would explain this cooperativity were not observed directly at the C2/3E6 binding interface, we hypothesized that cooperative binding may be the result of perturbation of either surface electrostatics or dynamics. Upon inspection of B factors for the fVIII C2 domain in each complex and in isolation, we determined that several loops distal to the 3E6 epitope displayed lower B factors relative to the entire C2 domain structure for each C2/3E6 complex. The associated decrease in mobility that is concomitant with lower B factors could decrease the entropic cost of binding a second, non-classical antibody, a hallmark of the induced fit binding mechanism often observed for antigen-antibody interactions^42^,^43^. While these data seem convincing that the cooperativity is a dynamic effect, they do not completely rule out electrostatic contributions. It should be noted that the 3E6 binding site sequesters the region of the fVIII C2 domain with the highest density of positive charge^28^,^38^. Given that both the 3E6 and G99 antibodies recognize regions of positive charge within significant portions of their respective epitopes, sequestering one binding site may allow for electrostatic steering for the second antibody to bind. To conclude, understanding the nature of the anti-fVIII immune response will further our understanding of inhibitor development following fVIII replacement therapy, thus hopefully leading to the development of more robust, less immunogenic replacement products.

## Methods

### Cloning, expression and purification of proteins

Generation of purified fVIII C2 domain was performed as previously described^28^,^29^. The fVIII C2 domain was inserted into a pET15b expression vector containing an N-terminal His_6_ affinity tag with a thrombin cleavage site. This expression construct was transformed into *Escherichia coli* NiCo21 cells (a BL21 (DE3) derivative) and subsequently grown at 37 °C in LB media in the presence of ampicillin to an OD_600_ of 0.6–0.8. Protein overexpression was induced upon the addition of isopropyl β–D-thiogalactopyranoside to 0.5 mM with adjustment of the incubation temperature to 15 °C for 16–20 hours. Overexpressed cell cultures were centrifuged at 8,000 rpm for 10 minutes at 4 °C (FIBERLite F10-6 × 500y rotor, Thermo Fisher Scientific), and the resultant cell pellet was resuspended in lysis buffer (20 mM Tris-HCl (pH 7.0), 300 mM NaCl, 10 mM imidazole (pH 7.0), 0.01% (v/v) Triton X-100, and 2.5% (v/v) glycerol). Resuspended cells were lysed by the addition of 1 mM PMSF and 0.75 mg/mL chicken egg white lysozyme for 15–20 minutes at 4 °C followed by sonication on ice with a ½-inch titanium horn attached to a Branson 450 sonifier (50% duty cycle) for two cycles of 1 minute. The fVIII C2 domain-containing cell lysate was centrifuged at 16-17,000 rpm for 30–35 minutes at 4 °C (FIBERLite F21-8 × 50y rotor, Thermo Fisher Scientific), and the supernatant was subsequently filtered with 5 μm and 0.45 μm cellulose syringe filters, sequentially. Filtered lysate was applied to TALON cobalt affinity resin (Clontech, Mountain View, CA), which was pre-equilibrated with lysis buffer, and incubated for 1 hour at 4 °C. The lysate/resin slurry was applied to a gravity flow column and washed with 10 column volumes (CV) of lysis buffer, 20 CV of wash buffer I (20 mM Tris-HCl (pH 7.2), 300 mM NaCl, 10 mM imidazole, 2.5% (v/v) glycerol), 10 CV of wash buffer II (20 mM Tris-HCl (pH 7.2), 150 mM NaCl, 10 mM imidazole, 2.5% (v/v) glycerol). Lastly, the hexahistidine-tagged fVIII C2 domain was eluted with 20 mM Tris-HCl (pH 7.2) 150 mM NaCl, 150 mM imidazole (pH 7.0), and 2.5% (v/v) glycerol, which was immediately dialyzed into ion exchange buffer (25 mM Tris-HCl (pH 7.2), 50 mM NaCl). The initial purified fraction of the fVIII C2 domain was further purified by ion exchange chromatography with a Macro-Prep^TM^ strong cation exchange column (Bio-Rad), through a salt gradient from 50 to 500 mM NaCl. The eluted C2 domain was concentrated to 6–8 mg/mL and a final purification step was completed by size exclusion chromatography with a Superdex 75 column (GE Healthcare), which was equilibrated ion exchange buffer.

The murine monoclonal hybridoma for the 3E6 antibody was generated and large-scale antibody productions were performed previously^28^,^29^. The 3E6 mAb was purified from hybridoma supernatant with the NAb^TM^ Protein A Plus spin column according to the manufacturer’s instructions (Thermo Scientific). The 3E6 F_AB_ fragments were subsequently cleaved with immobilized papain (Thermo Scientific) and further isolated by an additional Protein A spin column step to remove the Fc regions of the IgG. The resultant F_AB_ fragments were further purified by size exclusion chromatography with a Superdex 75 column (GE Healthcare) and concentrated to 10–20 mg/mL. The C2 domain/3E6 F_AB_ fragment binary complex was formed by incubation at 37 °C for 30 minutes with a 1.5-fold molar excess of the C2 domain. The C2/3E6 binary complex was then separated with a Superdex 75 column, concentrated to 5–10 mg/mL, flash frozen in liquid nitrogen and stored at −80 °C for crystallization trials.

### Crystallization, data collection and structure determination

Initial crystallization conditions were first identified following the manual setup of 24-well sparse matrix screens by hanging drop vapor diffusion. Crystals suitable for diffraction studies were grown by a 1:1 ratio of 8 mg/mL C2/3E6 binary complex with 10 mM MES (pH 6.4-6.8) and 20% (w/v) PEG 8000. Small, disordered crystals were grown within the first 7–9 days, diffracting to 3.2 Å resolution while larger crystals that diffracted to 2.6 Å resolution grew over the period of one year. Cryoprotection of crystals was performed by the iterative transfer of crystals to a drop containing 10 mM MES (pH 6.5), 22% PEG 8000, and 10–30% dimethyl sulfoxide, and the crystals were subsequently flash-frozen in liquid nitrogen for cryogenic X-ray data collection. X-ray diffraction data were collected to 2.6 Å resolution on a Rigaku Micromax-007HF rotating anode with Confocal Varimax Optics and an RAXIS-IV++ imaging plate detector at the Fred Hutchinson Cancer Research Center (Seattle, WA). Diffraction data were collected with CrystalClear (Rigaku) and indexed, integrated and scaled with HKL2000^44^. Phasing was accomplished by molecular replacement with the program PHASER as incorporated into the PHENIX crystallographic software suite^45^. The search models employed for molecular replacement were the isolated C2 domain (PDB: 1D7P), the 3E6 F_AB_ constant domain (PDB: 4KI5), and the 3E6 F_AB_ variable domain, which were searched for iteratively. Model building and refinement of the X-ray crystal structure were performed with COOT and PHENIX, respectively^45^,^46^. Validation of the final model from refinement was completed with Molprobity^47^. Calculations to determine pKa values were performed with PROPKA Version 3.0^48^. Small angle X-ray scattering (SAXS) data were collected at the SIBYLS beamline and processed with the ATSAS software suite^49^,^50^. Bead models resulting from DAMMIN/DAMMIF ab initio calculations were converted to molecular envelopes with Situs, and rigid body alignment of the C2/3E6 binary structures into the SAXS-derived molecular envelopes was performed in Chimera^51^,^52^.

## Additional Information

**Accession Numbers**: Model coordinates and structure factor amplitudes for the X-ray crystal structure of the factor VIII/3E6 binary complex were deposited in the Protein Data Bank (acc. #: 4XZU).

**How to cite this article**: Wuerth, M. E. *et al.* Structure of the Human Factor VIII C2 Domain in Complex with the 3E6 Inhibitory Antibody. *Sci. Rep.* **5**, 17216; doi: 10.1038/srep17216 (2015).

## Supplementary Material

Supplementary Information

## Figures and Tables

**Table 1:** Crystallographic data and refinement statistics.

Data Collection Statistics	
Wavelength (Å)	1.54
Resolution range (Å)	39.77–2.609 (2.702–2.609)
Space group	P 2 2_1_ 2_1_
Unit cell (Å, °)	a = 43.23, b = 148.49, c = 188.42, α = β = γ = 90
Total reflections	211,017
Unique reflections	36,886 (3,537)
Multiplicity	5.7 (5.8)
Completeness (%)	96.86 (93.47)
Mean I/sigma(I)	15.20 (6.08)
Wilson B-factor	48.03
R-merge	0.067 (0.292)
Refinement Statistics	
Resolution (Å)	40–2.61 (2.68–2.61)
R-work	0.1988 (0.2500)
R-free	0.2673 (0.3491)
Number of non-hydrogen atoms	9,034
macromolecules	8,790
ligands	3
water	241
Protein residues	1,137
RMS bonds (Å)	0.011
RMS angles (°)	1.36
Ramachandran	
favored (%)	91
allowed (%)	8.2
outliers (%)	0.8
*MolProbity* Clashscore	12.62
Average B-factor (Å^2^)	49.4
macromolecules	49.6
ligands	70.1
solvent	41.1
PDB code	4XZU
