# Supplementary material for: Structure of the Human Factor VIII C2 Domain in Complex with the 3E6 Inhibitory Antibody
Source: Sci Rep. 2015 Nov 24;5:17216. doi: 10.1038/srep17216 (PMC4657047; doi:10.1038/srep17216)
Supplement: Supplementary Information [file srep17216-s1.pdf]

Supplementary Materials

Structure of the Human Factor VIII C2 Domain in Complex with the 3E6 Inhibitory Antibody

Michelle E. Wuerth, Rebecca K. Cragerud and P. Clint Spiegel, Jr.*

Supplementary Figure S1

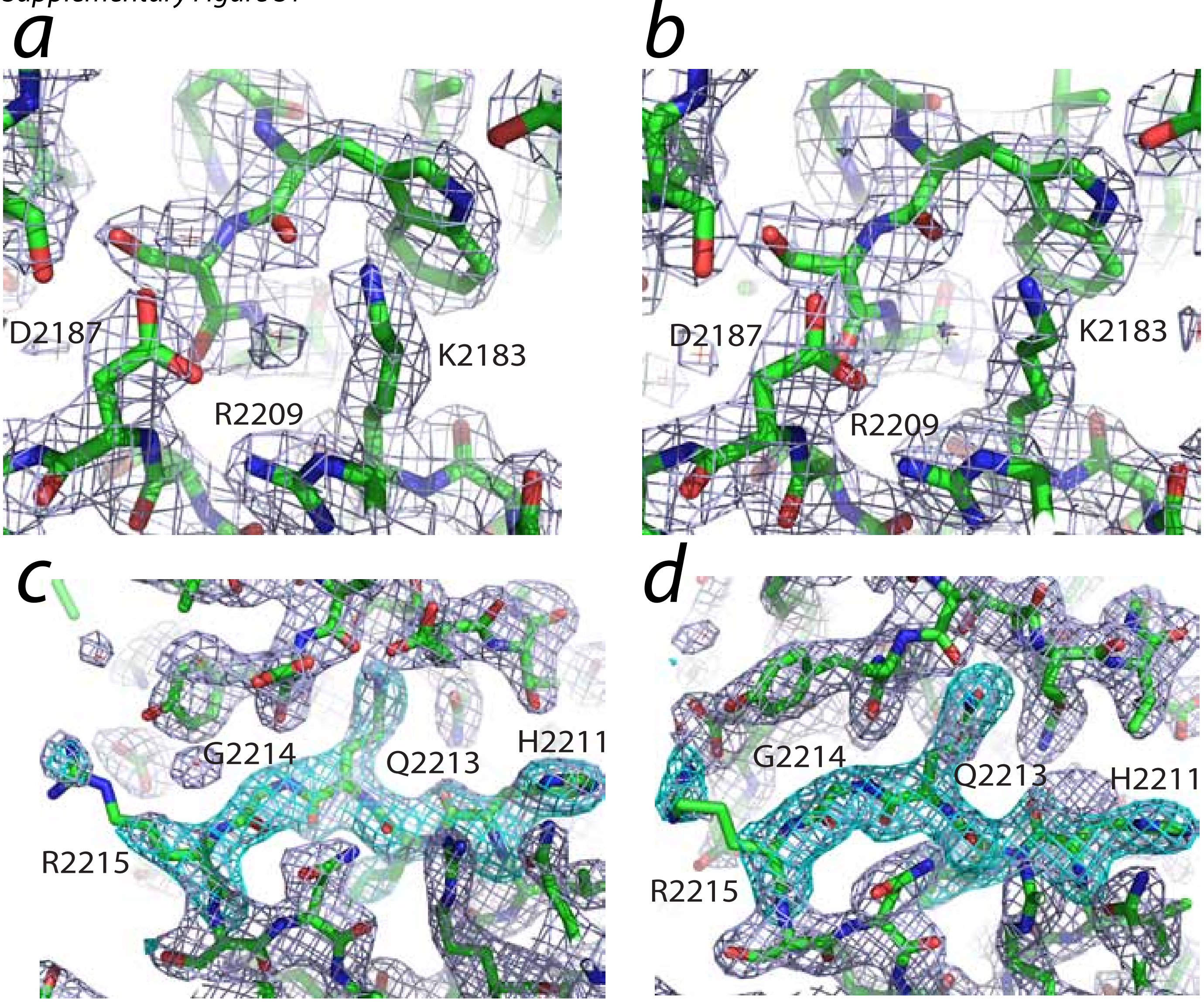

Electron density representation of the factor VIII C2 domain/3E6 FAB binding interface.

(a.b) The Glu2181-Ala2188 loop for binary complexes 1 and 2, respectively. Both Lys2183 and Asp2187 form conserved interactions with the 3E6 variable domain and are represented by positive electron density (2fo-fc).

(c,d) The Thr2202-Arg2215 loop for binary complexes 1 and 2, respectively. Conserved interactions are present for His2211, Gln2213 and Gly2214 with different conformations for Arg2215, all represented by positive electron density (2fo-fc: light blue, 1 sigma; Δ2211-2215 fo-fc: cyan, 3 sigma)
